# Protective effects of baicalin against deoxynivalenol-induced oxidative and inflammatory damage in chicken-derived hepatic 3D cell cultures

**DOI:** 10.1038/s41598-025-95868-0

**Published:** 2025-04-01

**Authors:** Júlia Vörösházi, Máté Mackei, Csilla Sebők, Patrik Tráj, Rege Anna Márton, Zsuzsanna Neogrády, Gábor Mátis

**Affiliations:** 1 Division of Biochemistry, Department of Physiology and Biochemistry, University of Veterinary Medicine, Budapest, 1078 Hungary; 2 National Laboratory of Infectious Animal Diseases, Antimicrobial Resistance, Veterinary Public Health and Food Chain Safety, University of Veterinary Medicine, Budapest, 1078 Hungary

**Keywords:** Biochemistry, Cell biology, Molecular biology

## Abstract

Deoxynivalenol (DON) is a trichothecene mycotoxin often contaminating grains used in poultry feed production and causing several adverse effects in farm animals. Therefore, it is important to investigate compounds that can be potential candidates to mitigate these effects, such as baicalin. The effects of DON and baicalin were investigated in chicken-derived 3D hepatic cell cultures, and cell viability, LDH activity, oxidative parameters (NRF-2, 8-OHdG) and inflammatory parameters (IL-6, IL-8, IFN-γ) were monitored for 24 and 48 h. Our results suggest that DON reduced cellular metabolic activity but did not prove to be cytotoxic, and baicalin was able to attenuate this adverse effect. The change in extracellular LDH activity suggests that after 48 h the cells have already started to respond to the adverse effects of the toxin and protective mechanisms were induced. Based on the measured oxidative parameters, baicalin showed antioxidant activity, but after longer exposure, our results indicate a prooxidant effect. Baicalin also had an anti-inflammatory effect based on the amount of IL-6 and IL-8, while DON exerted a dose-and time-dependent pleiotropic activity. These results suggest that DON may have an impact on cellular inflammation and oxidative homeostasis, and that baicalin could be able to alleviate these adverse effects.

## Introduction

Mycotoxins are secondary metabolites produced by fungi, which pose a significant concern in food and agricultural industries^1,2^. Consequently, there is a growing interest in controlling mycotoxin contamination of food and feed, as well as in understanding and ameliorating the adverse effects of mycotoxins in both humans and animals^1^. Trichothecenes, a prominent group of mycotoxins, are mainly produced by various *Fusarium* species that are found frequently on cereal grains^3,4^. Cool temperatures and high humidity are favorable for the fungal growth in the field, and infection can also occur under inadequate storage conditions, such as high moisture^5,6^. Over 170 trichothecene compounds have been identified, the majority of which can bind to the eukaryotic ribosome and inhibit protein synthesis^7,8^. The common element in their structure is the tetracyclic, sesquiterpenoid 12,14-epoxytrichothec-9-ene ring system, in which the epoxide group is responsible for the toxicity^8^. Trichothecenes can be classified into four groups based on the substitution patterns: type A, B, C and D^9^.

One of the most common representatives of type B trichothecenes is deoxynivalenol (DON, vomitoxin) which mainly contaminates wheat, maize, barley, oat, and rye^1,4,8,10^. DON, like other type B trichothecenes, has a keto group at C8^9^. It is predominantly produced by *F. graminearum* and *F. culmorum*, both of which can survive on leaves during the cold season and can serve as a source of infection for the following crop^4^. On average, poultry feed contains 0–5 mg/kg of DON, although occasionally this amount can be higher^4,5^. Avian species are typically less sensitive to the adverse effects of the toxin compared to mammals, probably due to the different absorption, distribution, metabolism, and elimination^11^. However, long-term exposure to DON can still have serious negative impacts in poultry^12,13^. The main effects of the toxin are reduced growth, feed refusal, immune modulation, gastrointestinal lesions, as well as neurological and reproductive damage^14^. The most important cellular action of the toxin is the inhibition of protein synthesis through the binding of the 60S subunit of the ribosome^4,15^. This results in ribosomal conformational changes that affect the peptidyl transferase activity^4^. DON also inhibits the synthesis of DNA and RNA and can alter cell membrane structure^4^. In addition, DON activates mitogen-activated protein kinases (MAPKs) which upregulate the expression of proinflammatory cytokines and chemokines, as well as induce apoptosis^16^.

Reactive oxygen species (ROS)-mediated oxidative stress induced by DON has also been reported in several studies, which may further contribute to its cytotoxic effect^16–18^. ROS are generated under normal physiological conditions, but their production must be balanced by the cellular antioxidant sytems. When this balance is disrupted, oxidative stress can occur, resulting in DNA damage, inflammation, as well as mitochondrial dysfunction^19,20^.

Nuclear factor erythroid 2-related factor 2 (NRF-2) is a key regulator of antioxidant, anti-inflammatory, and cytoprotective gene expression^21,22^. Under normal conditions, it is localized in the cytoplasm, bound to Kelch ECH associating protein 1 (Keap1)^23^. Upon oxidative stress, NRF-2 translocates to the nucleus, where it binds to the antioxidant response element (ARE) and regulates the expression of genes involved in the antioxidant response, including heme oxygenase-1 (HO-1), glutathione S-transferase (GST) and glutathione peroxidase^21,24,25^. Additionally, NRF-2 also influences inflammatory processes via the HO-1 axis^26^.

ROS production stimulated by DON can also damage DNA, and the presence of 8-hydroxy-2'-deoxyguanosine (8-OHdG) could be a good indicator of this impairment^27,28^. During DNA damage, hydroxyl radicals attack the DNA strand, forming C8-hydroxyguanine (8-OHGua) or its nucleoside form, 8-OHdG^28^. This is often converted to 8-oxo-7,8-dihydro-2'-deoxyguanosine (8-oxodG) by keto-enol tautomerism^27^.

Additionally, the binding of DON to the ribosomal subunit triggers a ribotoxic stress response, which can induce the production of pro-inflammatory cytokines and apoptosis by activating MAPKs^29^. Moreover, DON-induced oxidative stress can also affect the immune system in a dose- and exposure-dependent manner. It influences cellular cytokine production, including the secretion of interleukin (IL)-1β, -8, -6, -12, -18, tumor necrosis factor-α (TNF-α), and interferon-γ (IFN-γ)^30,31^. Furthermore, oxidative stress-induced inflammation is likely to be mediated through activation of the nuclear factor kappa B (NF-κB) pathway^29,30,32,33^. NF-κB is a key regulator of both the immune system and the inflammatory response, and it is also involved in cell proliferation and programmed cell death^29^. It has been hypothesized that NRF-2, together with the NF-κB signaling pathways cooperate to maintain physiological redox homeostasis and regulate the cellular inflammatory response, but the exact mechanism of this cooperation is not yet fully understood^26,34^.

Baicalin (BAI), a flavonoid compound and the glucuronide form of baicalein, can be found in the root of *Scutellaria baicalensis* Georgi^19,21,35^. It is known to exert several beneficial effects, including anti-inflammatory, antioxidant, antitumor, antimicrobial, and antiviral activities, which are widely used in therapeutic applications in many Eastern Asian countries^19,21^. On a cellular level, baicalin is able to suppress NF-κB, whose overexpression leads to the excess of pro-inflammatory mediators^36,37^. It also affects NRF-2 function through activation of the NRF2/HO-1 signaling pathway^36,38^.

In this study, the adverse effects of DON and the possible protective role of baicalin were investigated on chicken-derived three-dimensional (3D) hepatic cell cultures created by magnetic bioprinting, as a proper tool for mimicking the in vivo conditions of the avian liver. The effects of DON and baicalin on cellular viability, oxidative homeostasis, and inflammatory processes, as well as the potential of baicalin as a possible protective agent in DON-treated cells, were addressed.

### Animal welfare statement

All animal procedures were carried out in accordance with international and national laws, as well as the institutional policies. The study was approved by the Government Office of Zala County, Food Chain Safety, Plant Protection, and Soil Conservation Directorate, Budapest, Hungary (permission number: ZAI/040/00,522–7/2020). The study was conducted following the ARRIVE guidelines 2.0 (https://arriveguidelines.org/). The animals were fed and reared in accordance with the requirements of the Ross Technology^39^.

## Materials and methods

### Cell isolation and culturing conditions

All reagents used were purchased from Merck KGaA (Darmstadt, Germany) unless specified otherwise. Cells were isolated from three-week-old Ross 308 male broiler chicken (obtained from Gallus Poultry Farming and Hatching Ltd, Devecser, Hungary) following a previously described protocol by our research group^40^. Briefly, after narcosis with CO_2_, the animal was decapitated, and the abdominal cavity was opened to access the liver. Hepatic cells were harvested via a three-step perfusion through the *vena gastropancreaticoduodenalis*. The perfusion process applied collagenase to detach the hepatic cells and all buffers were preheated to 40 °C and freshly oxygenated with Carbogen (95% O_2_, 5% CO_2_). The perfusion rate was set to 30 mL/min.

The liver was first washed with 150 mL of 0.5 nM ethylene glycol tetraacetic acid (EGTA)-containing Hanks’ Balanced Salt Solution (HBSS), followed by a rinse with 150 mL EGTA-free HBSS. In order to release the cells, the extracellular matrix had to be disrupted using 130 mL of 7 mM MgCl_2_ and CaCl_2_ containing HBSS supplemented with 1 mg/mL type IV collagenase (Nordmark, Uetersen, Germany).

After the perfusion, the Glisson’s capsule was opened, and the obtained cells were resuspended in 50 mL ice-cold bovine serum albumin (BSA, 2.5%)-containing HBSS. Cell aggregate residues and undigested interstitium were removed by filtration through three layers of sterile gauze. The resulting filtrate was incubated on ice for 50 min.

Hepatocytes and non-parenchymal (NP) cells were separated by differential centrifugation. The suspension was centrifuged three times for 3 min at 100 × g. At each step, the NP cell-containing supernatant was collected separately, and the hepatocyte-containing sediment was resuspended in William’s Medium E supplemented with 0.22% NaHCO_3_, 50 mg/mL gentamycin, 2 mM glutamine, 4 µg/L dexamethasone, 20 IU/L insulin and 5% foetal bovine serum (FBS). At the end of the process, an NP cell-free, hepatocyte-rich fraction was obtained.The separately collected NP cell-containing supernatant was then centrifuged at 350 × g for 10 min to remove residual hepatocytes and red blood cells. The suspension was then centrifuged at 800 × g for 10 min, after which the sediment contained the NP cells.

Cell viability and total cell number were determined in Bürker chamber, using trypan blue staining. The number of viable cells for both cell types exceeded 90%. The cell number of hepatocyte-NP cell mixed suspension was then set to 5 × 10^5^ cells/mL, and the hepatocyte to NP cell ratio was 6:1 (to mimick a milder hepatic inflammation)^40,41^.

Materials and equipment for the magnetic 3D bioprinting were purchased from Greiner Bio-One Hungary Ltd (Mosonmagyaróvár, Hungary). First, 800 µL of magnetic nanoparticles (NanoShuttle™-PL) were added to 8 mL of cell suspension (according to the manufacturer’s instructions), then the cells were seeded onto 96-well cell-repellent plates and incubated for 1 h at 37 °C. During this time, the nanoparticles bound to the cell membranes and magnetized them. The plate was then transferred onto a Spheroid Drive, which contained small magnets positioned under each well of the plate, and incubated for 48 h at 37 °C in a humid atmosphere with 5% CO_2_. After 24 h, a medium exchange was also performed using a Holding Drive.

### Treatment of the cell cultures

**Fig. 1:**
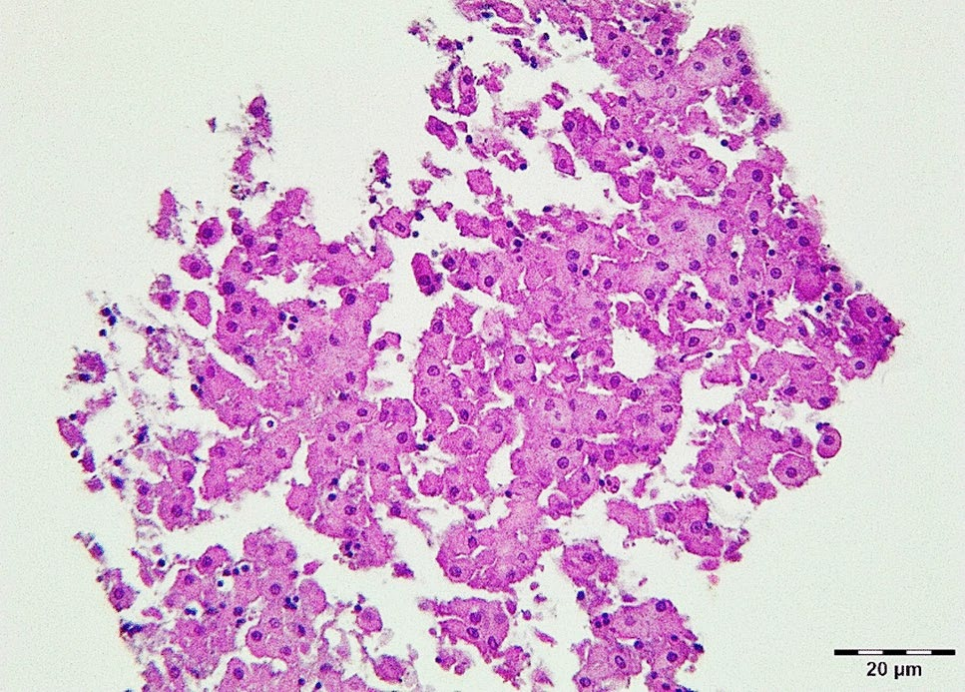
Primary 3D hepatic spheroid from chicken origin, randomly selected from control, untreated cells. The section was stained with hematoxylin and eosin (H&E) staining, bar: 20 µm. Well-preserved hepatocytes with intact morphology are present in high abundance, loosely connected to each other and to other NP cells with smaller nuclei. No significant degenerative changes are visible.

After 48 h of cell culturing, cells were treated with three concentrations of baicalin (95% purity; 5, 15, and 45 µg/mL) and two concentrations of DON (2 and 20 µg/mL), either alone or in combinations, for 24 and 48 h, as shown in Table 1. Following the incubations, the cell culture media was collected. After 48 h, the cells were lysated by intermittent sonication (1/second) in 40 µl M-PER (Mammalian Protein Extraction Reagent, Thermo Fisher SSC, Budapest, Hungary) buffer using Bandelin Sonopuls HD 2200 homogenizer (Bandelin Electronic GmbH & Co. KG, Berlin, Germany). The samples were then stored at -80 °C until further use. Some of the control, untreated spheroids were fixed in 10% buffered formalin solution, and after embedding and sectioning the slides they were stained with haematoxylin and eosin (H&E) to examine the spheroid morphology (Fig. 1). The process from isolation to the various measurements is shown schematically in Supplementary Fig. 1.

**Table 1:** Setting of the treatment groups.

Group	DON concentration	BAI concentration
Control	–	–
BAI5	–	5 µg/mL
BAI15	–	15 µg/mL
BAI45	–	45 µg/mL
DON2	2 µg/mL	–
DON20	20 µg/mL	–
DON2 + BAI5	2 µg/mL	5 µg/mL
DON2 + BAI15	2 µg/mL	15 µg/mL
DON2 + BAI45	2 µg/mL	45 µg/mL
DON20 + BAI5	20 µg/mL	5 µg/mL
DON20 + BAI15	20 µg/mL	15 µg/mL
DON20 + BAI45	20 µg/mL	45 µg/mL

BAI: baicalin, DON: deoxynivalenol.

### Metabolic activity of the cells

The metabolic activity of the cells was determined by Cell Counting Kit-8 (CCK-8) assay (Dojindo Molecular Technologies, Rockville, MD, USA). The measurement was performed according to the manufacturer’s protocol. The CCK-8 test is a colorimetric assay to measure the amount of reduced coenzymes produced during catabolic processes, and from this, the viability of the cells can be determined. In this method, 10 µL of CCK-8 reagent was added to 100 µL of cell culture medium, and after 2 h of incubation, the absorbances were read at 450 nm using a Multiskan GO 3.2 reader (Thermo Fisher Scientific, Waltham, MA, USA).

### Lactate dehydrogenase activity of the cells

To investigate the cell membrane damage, the activity of extracellular lactate dehydrogenase (LDH) was measured using the LDH Activity Assay Kit. Following the manufacturer’s instructions, 50 µL of Master Reaction Mix (48 µL LDH Assay Buffer, 2 µL LDH Substrate Mix) was added to 50 µL of sample, and then the absorbances were read at 450 nm using a Multiskan GO 3.2 reader. To determine the LDH activity, the absorbances were read every 5 min until the absorbance of the most active sample exceeded the absorbance of the most concentrated standard. The activity was then calculated using the equation in the manufacturer’s protocol.

### Cellular oxidative stress and inflammation

Oxidative parameters and inflammatory cytokines were quantified using chicken-specific ELISA kits (MyBioSource, San Diego, CA, USA) according to the manufacturer’s instructions. For the investigation of oxidative stress, NRF-2 was measured from cell culture medium (sensitivity is up to 5.7 pg/mL), and the level of 8-OHdG was determined from the cell lysates (sensitivity is up to 0.05 ng/mL). To monitor the inflammatory processes, the concentrations of IL-6 and -8, as well as IFN-γ (sensitivity is up to 5 pg/mL, 5.1 pg/mL, 5 pg/mL, respectively), were assessed from the cell culture medium.

### Statistical analysis

The statistical analysis was performed using R Statistical Software (R Core Team 2023). Each treatment group consisted of 8 replicates (n = 8/group). During the analysis, groups treated with baicalin or DON alone were compared to the control group, and groups exposed to the combination treatments were compared to the corresponding DON-treated groups. Shapiro–Wilk and Levene’s tests were performed to verify the normal distribution and homogeneity of variance. One-way analysis of variance (ANOVA) was used to compare differences between groups, and Dunett’s post hoc test was used for pairwise comparisons. Results were expressed as mean ± standard error of the mean (SEM). Differences were considered significant at *p* < 0.05. Results were visualized using Graphpad Prism version 9.1.2. for Windows (GraphPad Software, San Diego, CA, USA).

## Results

### Metabolic activity of the cells

**Fig. 2:**
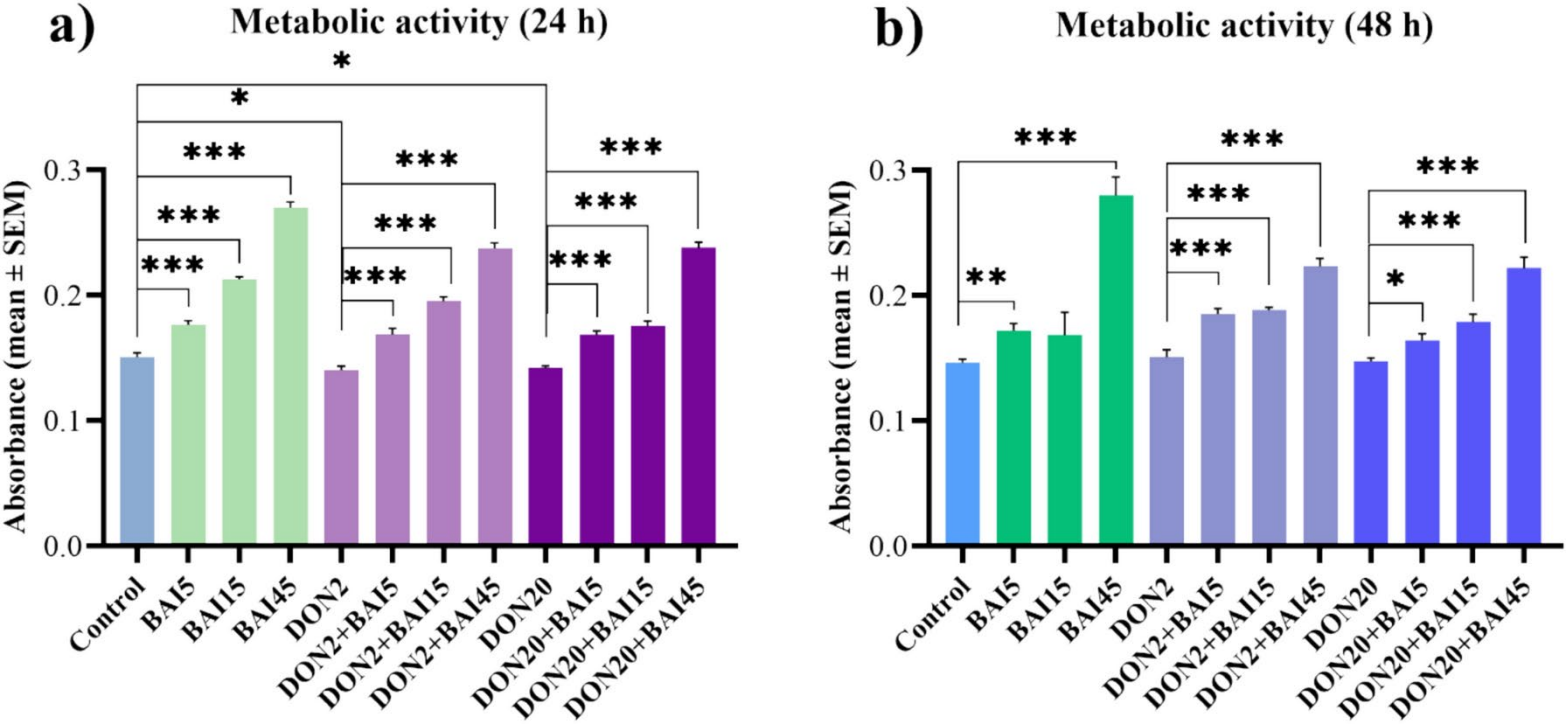
Effects of (**a**) 24 h and (**b**) 48 h of deoxynivalenol (DON) and baicalin (BAI) treatment on the metabolic activity of primary hepatic 3D cell co-cultures of chicken origin assessed by CCK-8 test. BAI and DON-treated cells were compared to the control group. Different baicalin and 2 µg/mL DON-treated cells were compared to the 2 µg/mL DON group. Different baicalin and 20 µg/mL-DON treated cells were compared to the 20 µg/mL DON group. The metabolic activity of the cells was overall increased by the baicalin treatment after 24 and 48 h, and decreased by the applied DON concentrations after 24 h. Control: cells without DON or BAI exposure; BAI: cells treated with BAI; DON: cells treated with DON; DON + BAI: cells treated with the combination of DON and BAI. See Table 1 for detailed concentrations. Results are expressed as mean ± SEM. *p < 0.05; **p < 0.01; ***p < 0.001.

The metabolic activity of the cells was measured by CCK-8 assay. No significant differences were observed between the control groups of different incubation times (0.151 ± 0.004 and 0.147 ± 0.003 for 24 and 48 h control groups, respectively, with *p* = 0.364). In the case of the 24 h incubation, all three baicalin concentrations significantly increased (*p* < 0.001 in all cases) the cellular viability compared to control (Fig. 2**/**a). In contrast, DON decreased (*p* = 0.042; *p* = 0.044, respectively) the metabolic activity at both used concentrations (Fig. 2**/**a). When combined with the 2 µg/mL DON treatment, all three baicalin treatments elevated (*p* < 0.001 in all cases) the viability of the cells compared to the cultures solely exposed to DON (Fig. 2**/**a). The same increasing trend (*p* < 0.001 in all cases) was observed when baicalin was combined with 20 µg/mL DON treatment (Fig. 2**/**a).

After the 48 h incubation, the 5 µg/mL and 45 µg/mL baicalin treatments significantly increased (*p* = 0.001; *p* < 0.001, respectively) the cellular viability in comparison with the absolute controls (Fig. 2**/**b), while DON exposure did not affect it. All three baicalin treatments in combination with 2 µg/mL DON elevated (*p* < 0.001 in all cases) the metabolic activity of cells compared to the DON-exposed controls (Fig. 2**/**b). Similarly, all three baicalin concentrations increased (*p* = 0.017; *p* < 0.001; *p* < 0.001, respectively) the cellular viability in combination with the 20 µg/mL DON treatment (Fig. 2**/**b). The mean ± SEM values obtained from the CCK-8 measurement and the corresponding significances are shown in Supplementary Table 1. The *p* values of the differences between the combination treatments and the control cell cultures are also included in Supplementary Table 1.

### Extracellular LDH activity

**Fig. 3:**
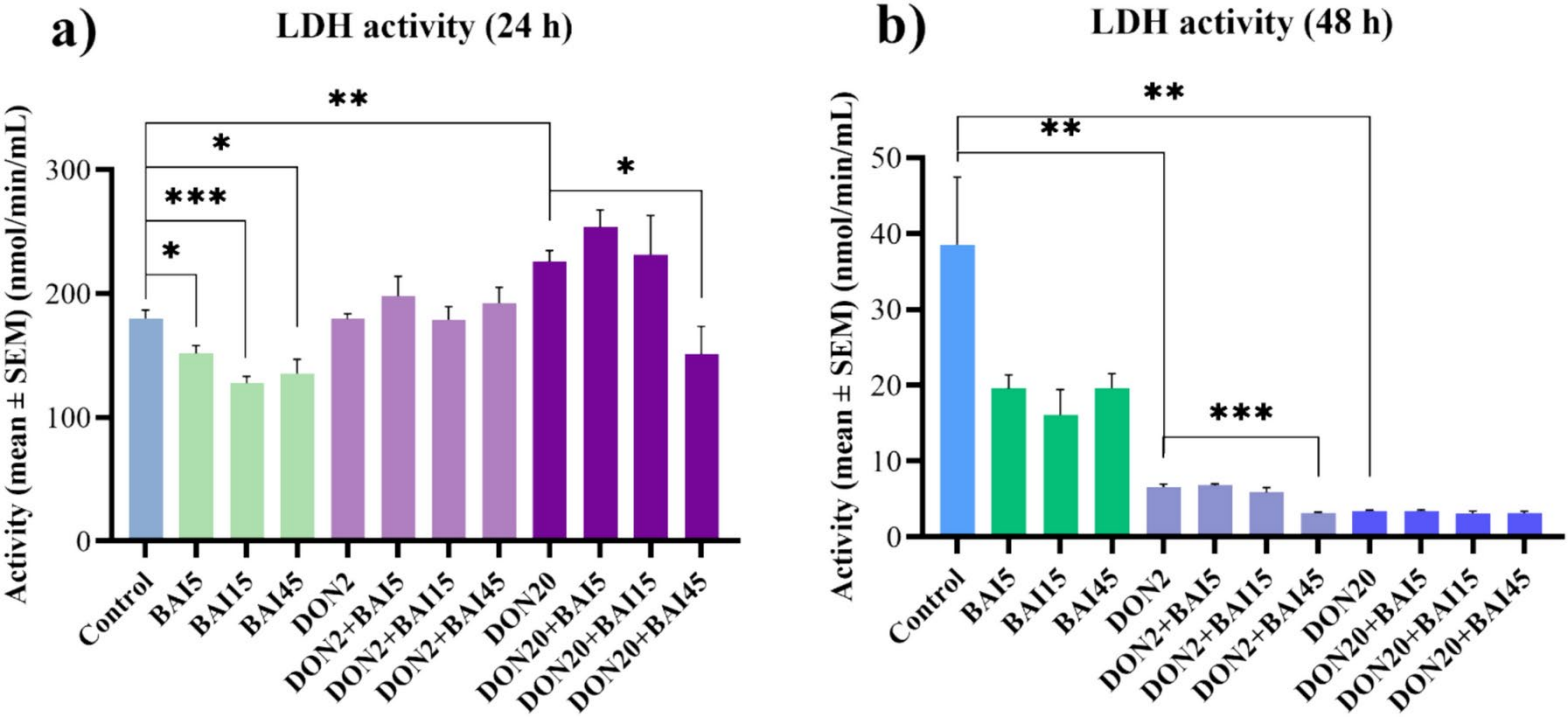
Effects of (**a**) 24 h and (**b**) 48 h of deoxynivalenol (DON) and baicalin (BAI) treatment on the LDH activity of primary hepatic 3D cell co-cultures of chicken origin assessed by LDH activity assay kit. BAI and DON-treated cells were compared to the control group. Different baicalin and 2 µg/mL DON-treated cells were compared to the 2 µg/mL DON group. Different baicalin and 20 µg/mL-DON treated cells were compared to the 20 µg/mL DON group. After 24 h treatment, the applied baicalin concentrations reduced, while the 20 µg/mL DON increased the LDH leakage of the cells. However, the 45 µg/mL baicalin mitigated this elevation. After 48 h, both DON treatments decreased the LDH activity, and the 45 µg/mL baicalin concentration further lowered it compared to the 2 µg/mL DON group. Control: cells without DON or BAI exposure; BAI: cells treated with BAI; DON: cells treated with DON; DON + BAI: cells treated with the combination of DON and BAI. See Table 1 for detailed concentrations. Results are expressed as mean ± SEM. *p < 0.05; **p < 0.01; ***p < 0.001.

To assess the membrane damage, extracellular LDH activity was measured from the medium of the cells. Upon incubation for 24 h, all three baicalin concentrations decreased (*p* = 0.017; *p* < 0.001; *p* = 0.010, respectively) and the 20 µg/mL DON increased (*p* = 0.003) the LDH leakage (Fig. 3**/**a). In addition, the 45 µg/mL baicalin treatment attenuated (*p* = 0.014) this DON-triggered elevated LDH activity (Fig. 3**/**a). In case of the 2 µg/ml DON treatment, no significant changes were observed (Fig. 3**/**a).

For the 48 h samples, both DON concentrations lowered (*p* = 0.005; *p* = 0.003) the LDH release of the cells (Fig. 3**/**b). Additionally, 45 µg/mL baicalin further decreased (*p* < 0.001) the LDH activity when applied together with 2 µg/mL DON (Fig. 3**/**b). However, when applied in combination with the 20 µg/ml DON, no significant changes were observed (Fig. 3**/**b). The mean ± SEM values obtained from the LDH activity measurement and the corresponding significances are shown in Supplementary Table 1. The *p* values of the differences between the combination treatments and the control cell cultures are also included in Supplementary Table 1.

### Concentration of oxidative parameters

**Fig. 4:**
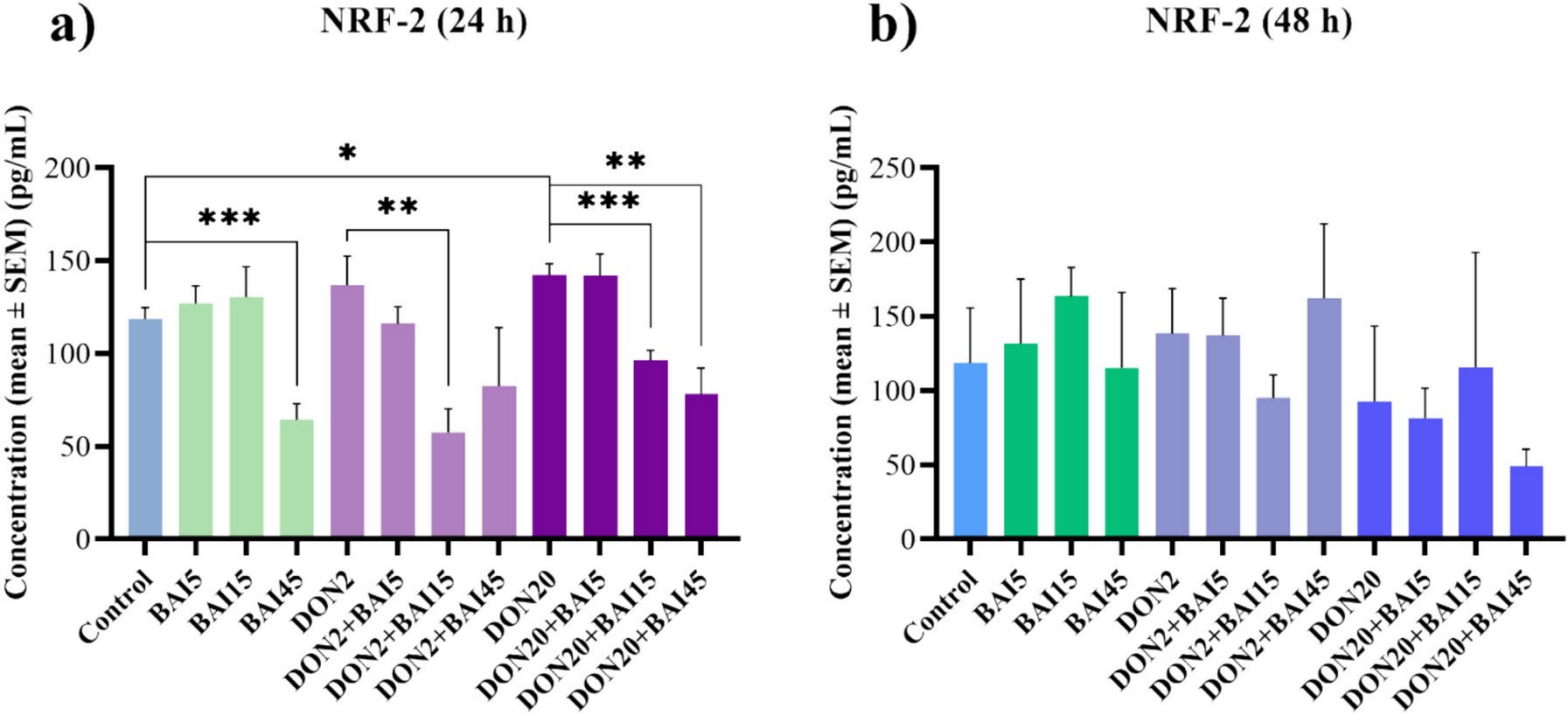
Effects of (**a**) 24 h and (**b**) 48 h of deoxynivalenol (DON) and baicalin (BAI) treatment on the NRF-2 content of primary hepatic 3D cell co-cultures of chicken origin assessed by chicken-specific ELISA test. BAI and DON-treated cells were compared to the control group. Different baicalin and 2 µg/mL DON-treated cells were compared to the 2 µg/mL DON group. Different baicalin and 20 µg/mL-DON treated cells were compared to the 20 µg/mL DON group. After 24 h, the 45 µg/mL baicalin concentration reduced the NRF-2 content, while the DON treatments elevated it. Additionally, the 15 µg/mL baicalin treatment decreased the NRF-2 level compared to the 20 µg/mL DON-treated group. Control: cells without DON or BAI exposure; BAI: cells treated with BAI; DON: cells treated with DON; DON + BAI: cells treated with the combination of DON and BAI. See Table 1 for detailed concentrations. Results are expressed as mean ± SEM. *p < 0.05; **p < 0.01; ***p < 0.001.

NRF-2 concentration was determined from the cell culture media using a chicken-specific ELISA assay. After 24 h of treatment, 45 µg/mL baicalin decreased (*p* < 0.001) and 20 µg/mL DON increased (*p* = 0.029) the NRF-2 content (Fig. 4**/**a). In addition, in combination with 2 µg/mL DON treatment, 15 µg/mL baicalin lowered (*p* = 0.007) the amount (Fig. 4/a). The elevated levels of NRF-2 by 20 µg/mL DON were attenuated (*p* < 0.001; *p* = 0.003, respectively) by the 15 µg/mL and 45 µg/mL baicalin treatment (Fig. 4**/**a). In contrast, after 48 h, no significant changes were observed (Fig. 4**/**b).

**Fig. 5:**
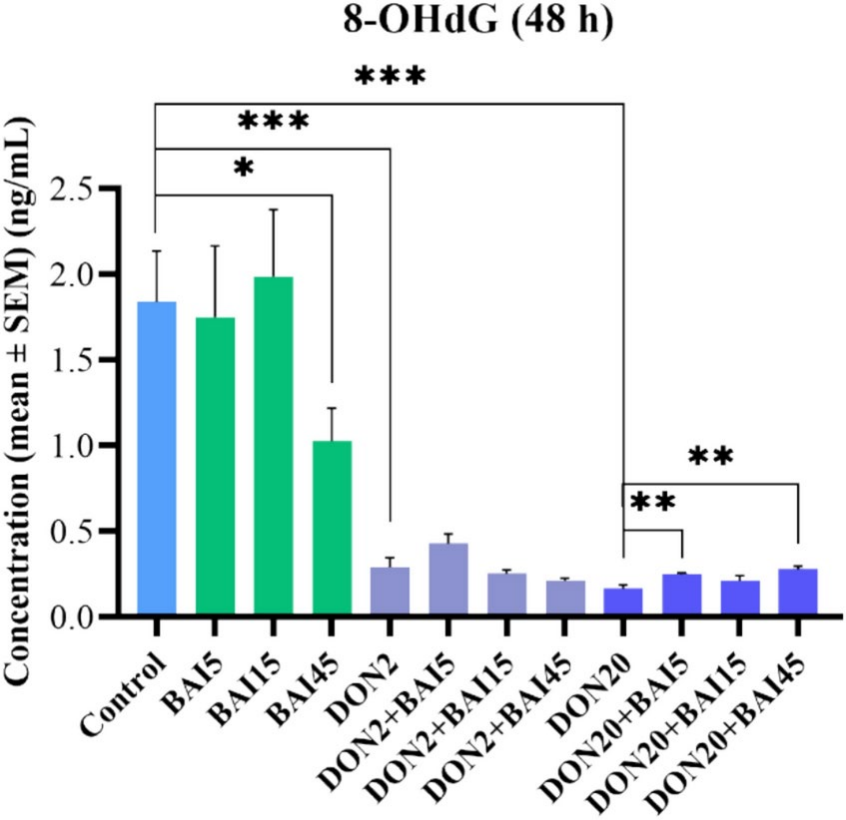
Effects of 48 h of deoxynivalenol (DON) and baicalin (BAI) treatment on the 8-OHdG content of primary hepatic 3D cell co-cultures of chicken origin assessed by chicken-specific ELISA test. BAI and DON-treated cells were compared to the control group. Different baicalin and 2 µg/mL DON-treated cells were compared to the 2 µg/mL DON group. Different baicalin and 20 µg/mL-DON treated cells were compared to the 20 µg/mL DON group. The 45 µg/mL baicalin, as well as both DON treatments, had an 8-OHdG-reducing effect on the cells. Control: cells without DON or BAI exposure; BAI: cells treated with BAI; DON: cells treated with DON; DON + BAI: cells treated with the combination of DON and BAI. See Table 1 for detailed concentrations. Results are expressed as mean ± SEM. *p < 0.05; **p < 0.01; ***p < 0.001.

The amount of 8-OHdG was measured from cell lysate after 48 h. The 45 µg/mL baicalin and both applied DON concentrations reduced (*p* = 0.049; *p* < 0.001; *p* < 0.001, respectively) the 8-OHdG concentration compared to the control group (Fig. 5). Furthermore, in combination with the 20 µg/mL DON treatment, the 5 and 45 µg/mL baicalin increased (*p* = 0.005; *p* = 0.004, respectively) the levels of 8-OHdG (Fig. 5). Regarding the 2 µg/ml DON concentration, the combination treatments did not result in significant changes (Fig. 5). The mean ± SEM values obtained from the NRF-2 and 8-OHdG measurements and the corresponding significances are shown in Supplementary Table 1. The *p* values of the differences between the combination treatments and the control cell cultures are also included in Supplementary Table 1.

### Concentration of inflammatory cytokines

**Fig. 6:**
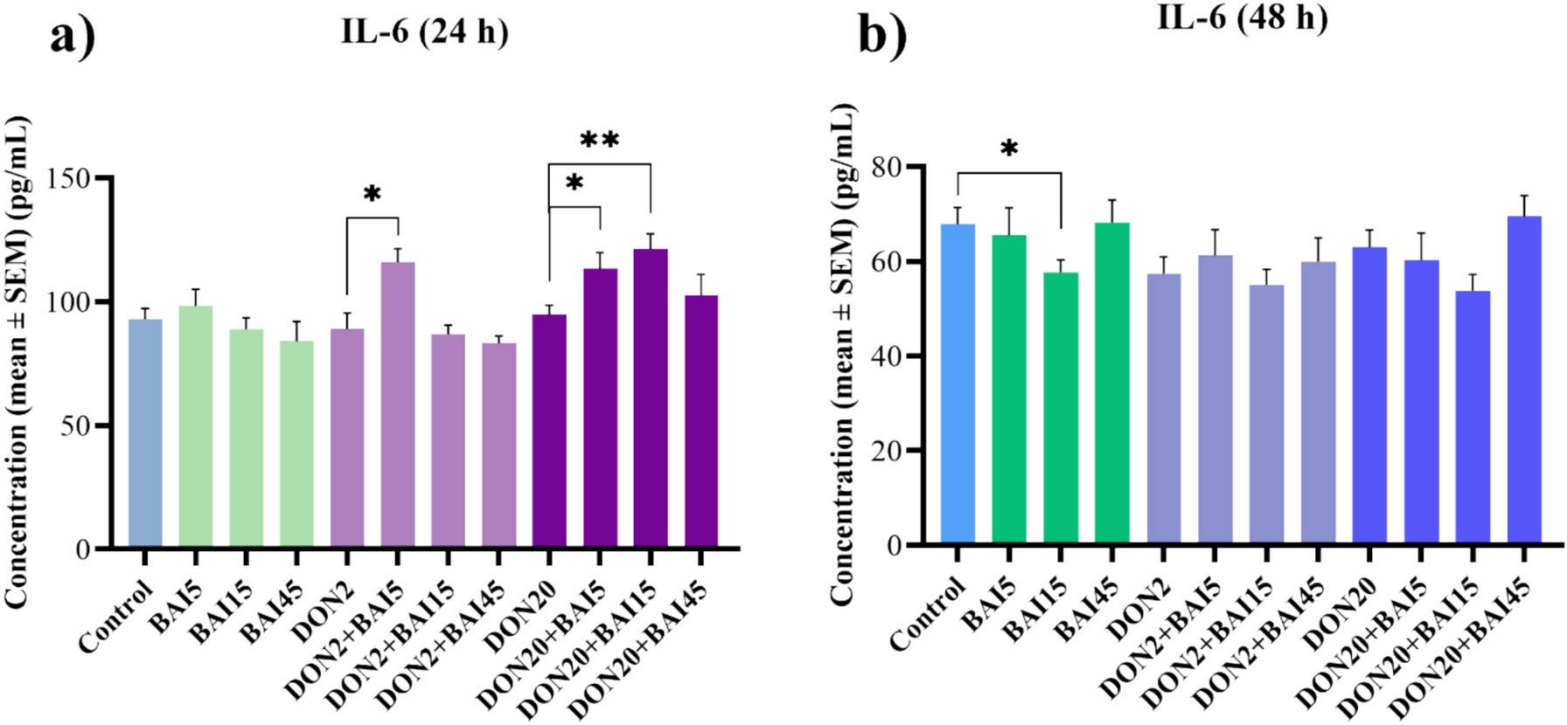
Effects of (**a**) 24 h and (**b**) 48 h of deoxynivalenol (DON) and baicalin (BAI) treatment on the IL-6 content of primary hepatic 3D cell co-cultures of chicken origin assessed by chicken-specific ELISA test. BAI and DON-treated cells were compared to the control group. Different baicalin and 2 µg/mL DON-treated cells were compared to the 2 µg/mL DON group. Different baicalin and 20 µg/mL-DON treated cells were compared to the 20 µg/mL DON group. After 24 h, the 5 µg/mL baicalin concentration elevated the IL-6 level in case of both DON treatments. Additionally, the applied 15 µg/mL baicalin also increased the amount of IL-6 compared to the 20 µg/mL DON group. After 48 h, the 15 µg/mL baicalin treatment reduced the IL-6 concentration. Control: cells without DON or BAI exposure; BAI: cells treated with BAI; DON: cells treated with DON; DON + BAI: cells treated with the combination of DON and BAI. See Table 1 for detailed concentrations. Results are expressed as mean ± SEM. *p < 0.05; **p < 0.01.

The inflammatory parameters were measured from the cell culture media using chicken-specific ELISA kits. DON did not affect the IL-6 levels after either incubation period. After 24 h of incubation, none of the treatments changed the levels of IL-6 compared to the control group (Fig. 6**/**a). However, the 5 µg/mL baicalin in combination with the 2 µg/mL DON treatment increased (*p* = 0.014) the IL-6 levels (Fig. 6**/**a). For the 20 µg/mL DON, the 5 and 15 µg/mL elevated (*p* = 0.039; *p* = 0.005, respectively) the amount of IL-6 (Fig. 6**/**a). Furthermore, after 48 h of treatment, the solely applied 15 µg/mL BAI lowered (*p* = 0.047) the IL-6 concentration compared to the control group (Fig. 6**/**b). However, no significant changes were observed in the case of the combination treatments (Fig. 6**/**b).

**Fig. 7:**
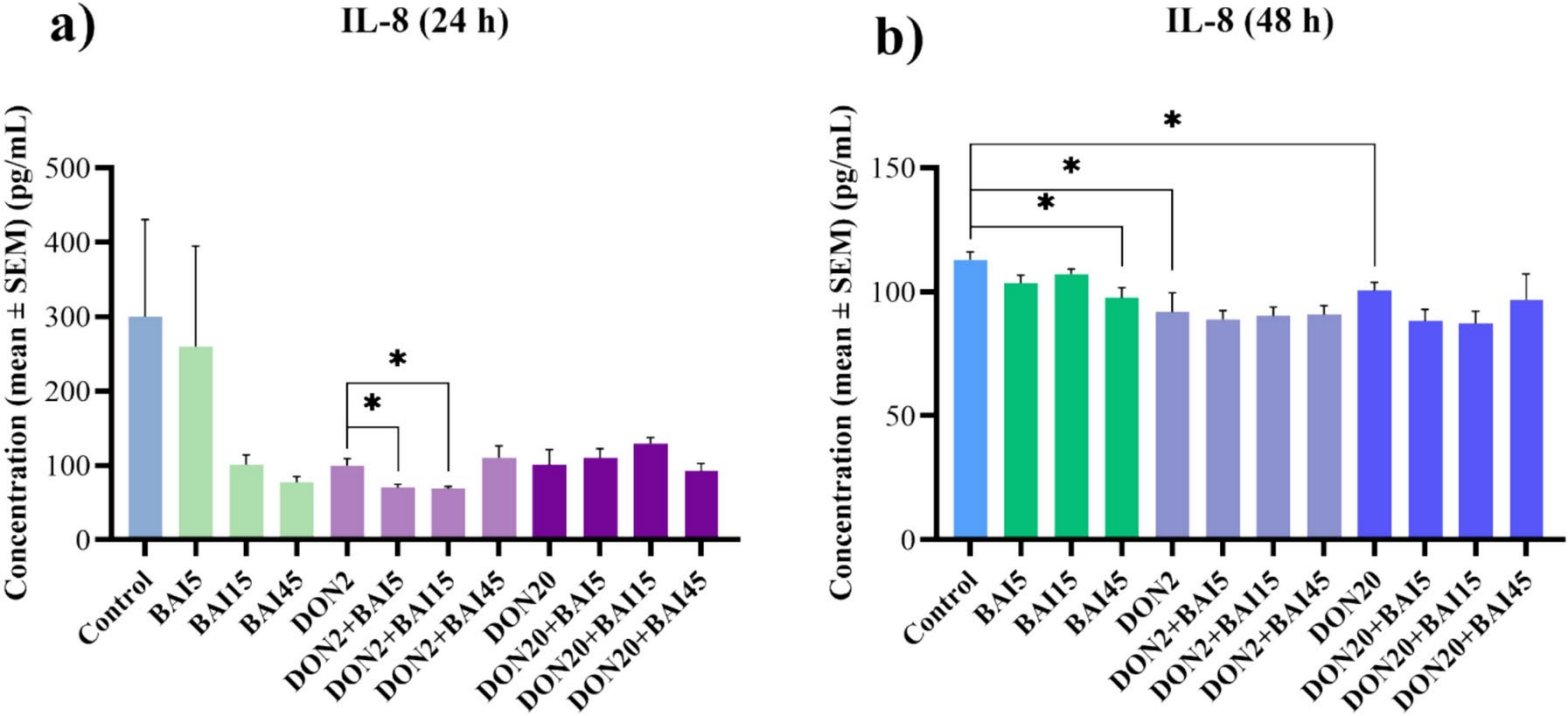
Effects of (**a**) 24 h and (**b**) 48 h of deoxynivalenol (DON) and baicalin (BAI) treatment on the IL-8 content of primary hepatic 3D cell co-cultures of chicken origin assessed by chicken-specific ELISA test. BAI and DON-treated cells were compared to the control group. Different baicalin and 2 µg/mL DON-treated cells were compared to the 2 µg/mL DON group. Different baicalin and 20 µg/mL-DON treated cells were compared to the 20 µg/mL DON group. After 24 h, both the 5 and 15 µg/mL baicalin concentrations lowered the amount of IL-8 compared to the 2 µg/mL DON group. After 48 h, the 45 µg/mL baicalin, as well as both DON treatments, reduced the level of IL-8. Control: cells without DON or BAI exposure; BAI: cells treated with BAI; DON: cells treated with DON; DON + BAI: cells treated with the combination of DON and BAI. See Table 1 for detailed concentrations. Results are expressed as mean ± SEM. *p < 0.05.

In case of the IL-8 content, after 24 h, DON had no influence on IL-8 levels by itself (Fig. 7**/**a). The 2 µg/mL DON treatment in combination with the 5 and 15 µg/mL baicalin reduced (*p* = 0.024; *p* = 0.016 respectively) the amount after 24 h (Fig. 7**/**a). However, no significant changes were observed in the case of the combination treatments with the 20 µg/ml DON (Fig. 7**/**a). After the 48 h incubation, the 45 µg/mL baicalin and both applied DON reduced (*p* = 0.016; *p* = 0.034; *p* = 0.024, respectively) the levels of IL-8 compared to the control (Fig. 7**/b**). Regarding the combination treatments, no significant changes were shown (Fig. 7**/**b).

**Fig. 8:**
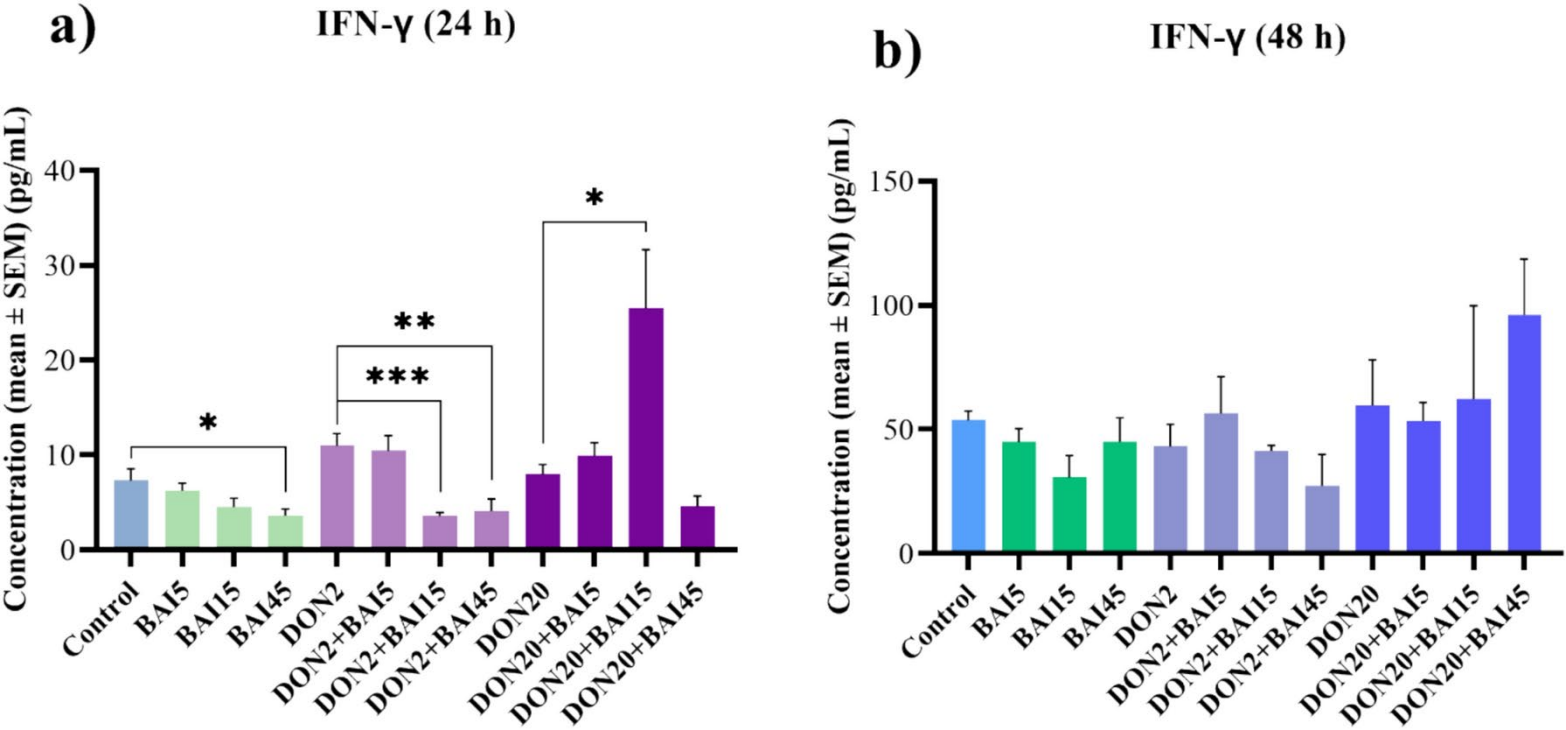
Effects of (**a**) 24 h and (**b**) 48 h of deoxynivalenol (DON) and baicalin (BAI) treatment on the IFN-γ content of primary hepatic 3D cell co-cultures of chicken origin assessed by chicken-specific ELISA test. BAI and DON-treated cells were compared to the control group. Different baicalin and 2 µg/mL DON-treated cells were compared to the 2 µg/mL DON group. Different baicalin and 20 µg/mL-DON treated cells were compared to the 20 µg/mL DON group. After 24 h, the 45 µg/mL baicalin reduced, while both DON treatments increased the amount of IFN-γ. Compared to the 2 µg/mL DON group, the 15 and 45 µg/mL baicalin treatment decreased, while compared to the 20 µg/mL DON group, the 15 µg/mL increased the IFN-γ concentration. Control: cells without DON or BAI exposure; BAI: cells treated with BAI; DON: cells treated with DON; DON + BAI: cells treated with the combination of DON and BAI. See Table 1 for detailed concentrations. Results are expressed as mean ± SEM. *p < 0.05; **p < 0.01; ***p < 0.001.

After 24 h, the 45 µg/mL baicalin reduced (*p* = 0.033) IFN-γ levels compared to the untreated cells (Fig. 8**/a**). In case of the 2 µg/mL DON treatment, the concomitantly administered 15 and 45 µg/mL baicalin diminished (*p* < 0.001 ; *p* = 0.005, respectively) the IFN-γ conentrations (Fig. 8**/**a). In addition, the 20 µg/mL DON in combination with the 15 µg/mL baicalin increased (*p* = 0.023) the amount of IFN-γ (Fig. 8**/**a). No significant changes were detected after the 48 h treatment (Fig. 8**/**b). The mean ± SEM values obtained from the IL-6, IL-8 and IFN-γ measurements and the corresponding significances are shown in Supplementary Table 1. The *p* values of the differences between the combination treatments and the control cell cultures are also included in Supplementary Table 1.

## Discussion

In this study, the effects of DON and baicalin were investigated in chicken-derived 3D liver cell cultures, that were created by magnetic 3D bioprinting. The principle of this method involves magnetizing cells with magnetic nanoparticles consisting of iron, poly-L-lysine, and gold. These nanoparticles electrostatically bind to the cell membranes via the poly-L-lysine at a concentration of approximately 50 pg/cell^48,49^. This amount can sufficiently magnetize the cells, allowing them to aggregate into spheroids when placed in a magnetic field^50^.The particles are biocompatible and do not interfere with cell growth and physiology^50,51^. Once magnetized, the cells can be arranged into 3D structures on multiwell plates using cylindrical magnets^52^. In this experiment, the hepatocyte to NP cell ratio was set to 6:1, mimicking a milder inflammation with moderate intrahepatic macrophage migration, which is suitable for modeling oxidative stress-induced and inflammatory processes^40,41^. The applied baicalin and DON concentrations have been set based on literature data related to cell culture studies^42–47^. Notwithstanding that the DON and baicalin concentrations used in the present study are higher than the supposed tissue levels correlating to average feed contamination, they are in line with previous in vitro studies, being suitable for monitoring the DON- and baicalin-associated hepatocellular molecular mechanisms^42–47^. As this is a limitation of the study and should certainly be taken into account in future in vivo experiments, we strongly advise examining a substantially lower range of DON and baicalin concentrations to mirror biologically occurring patterns.

Based on the results of the CCK-8 measurement, baicalin increased the metabolic activity and, therefore, the cell viability in all cases, applied either solely or in combination with DON, both compared to the control group and the different DON treatments, respectively. These findings confirm that baicalin generally has a positive effect on cell viability^19,21^. DON, on the other hand, decreased cell viability after 24 h of treatment. Since DON primarily inhibits protein synthesis, it is able to suppress catabolic processes in cells, leading to an energy-deficient state^4,13,15^. This aligns with previous findings reporting that DON reduces cell viability in a dose-dependent manner in various cell types, including bovine mammary epithelial cells, piglet hippocampal nerve cells, chicken splenic lymphocytes, and chicken embryo fibroblast^29,43,53,54^. However, this effect was not considered to be cytotoxic, as the rate of decrease was on average less than 10%. In our previous experiments, the effect of T-2 toxin, another trichothecene mycotoxin, was evaluated using the same cell culture model, which showed a more prominent decreasing effect on the cellular viability (on average 25%), though it did also not meet the threshold for cytotoxicity^13,55^. Additionally, there was no significant difference between the metabolic activity of the 24 and 48 h control groups, indicating that the duration of incubation did not influence the cellular viability. Notably, after 48 h, the viability-reducing effect of DON was absent, suggesting a potential metabolic adaptation in liver cells. This observation implies that the cells were able to recover after prolonged exposure, which is beneficial as it improves their capacity to respond to various stressors^56^.

The measurement of extracellular LDH activity serves as an indicator of membrane integrity, as LDH is released into the cell culture media upon membrane damage^57^. In this study, after 24 h, all three baicalin treatments had a positive, LDH-reducing effect compared to the control cells, suggesting an intact cell membrane. In contrast, treatment with the higher DON concentration significantly increased the LDH release, but this elevating effect was mitigated by the highest baicalin concentration in co-treated cells. This indicates that baicalin alleviated the DON-induced membrane damage of the cells. These findings are consistent with previous studies, where DON had an increasing effect on the extracellular LDH activity, while baicalin decreased it^29,58,59^. For instance, in a study by Liao et al., the intestinal protective effect of baicalin was investigated in DON-treated piglets. In this experiment, they found that the serum LDH activity of animals consuming DON-contaminated feed was significantly increased, which then was mitigated by baicalin^59^. Interestingly, however, after 48 h, both applied DON concentrations reduced the LDH leakage, and the highest dose of baicalin combined with the lower DON treatment further decreased the extracellular LDH activity. This observation suggests that mycotoxin-induced protective mechanisms were activated in the cells after 48 h DON exposure. These mechanisms potentially involve the initiation of autophagy, a process through which damaged cell organelles are degraded and eliminated^42,60,61^. Such responses might reflect a cellular attempt to overcompensate against the harmful effects of DON and seek to restore cellular homeostasis^13,55^.

The abundance of NRF-2, a key regulator in the antioxidant response, decreased with the highest baicalin concentration, whereas it increased with the higher DON treatment compared to untreated cells after the 24 h incubation. This suggests that DON induced oxidative stress in the cells, triggering an NRF-2-mediated cellular defence response, whereas baicalin appeared to exert an opposite antioxidant effect, potentially lowering NRF-2 levels and stabilizing the oxidative homeostasis.

For the lower DON treatment, the middle baicalin concentration decreased the amount of NRF-2, further indicating an antioxidant effect in the cells. However, this effect was not observed with the highest baicalin concentration. This phenomenon could be explained by the so-called non-monotonic dose response (NMDR). The basic hypothesis is that dose responses (DR) exhibit a dose-dependent monotonicity^62^. In NMDR, however, the direction of the DR curve changes in response to certain doses, although the specific conditions that lead to this are not yet fully understood^62–64^. In contrast, when baicalin was applied in combination with the higher DON concentration, both the middle and the highest baicalin treatments exhibited a dose-dependent, NRF-2 reducing antioxidant effect, demonstrating a monotonic DR in this case. Notably, after 48 h, NRF-2 levels were unaffected by either the baicalin or the DON treatments, consistent with the above-mentioned hypothesis that cells adapted to the exposure and restored their cellular homeostasis.

The amount of 8-OHdG, a marker of DNA oxidation, was assessed only after 48 h from the cell lysates. Compared to the control group, baicalin showed an antioxidant effect by reducing the amount of 8-OHdG. Interestingly, both applied DON treatments also exerted a reducing effect, potentially reflecting the activation of the overcompensatory defense mechanisms mentioned earlier^13,55^. However, when combined with the higher DON treatment, both the lowest and highest baicalin concentrations increased the amount of 8-OHdG. The fact that only these two concentrations had an elevating effect, while the middle one did not, can also be explained by the previously discussed NMDR theory. Moreover, the increasing effect of baicalin, rather than the expected alleviation, may be related to the dual role of flavonoids as both antioxidants and prooxidants, although the exact mechanism remains unclear^65,66^. The prooxidant effect of flavonoids is likely proportional to the number of hydroxyl groups in their structure. Flavonoids with fewer hydroxyl groups did not, whereas those with more hydroxyl groups—such as baicalin’s aglycone, baicalein – demonstrated prooxidant activity when tested by the Fenton reaction^67–69^. They are also able to reduce Cu(II) to Cu(I), initiating ROS production, and may also influence peroxidase enzymes that catalyse the oxidation of polyphenols^70,71^. Due to these prooxidant effects, they are even able to cause oxidative damage by reacting with lipids, proteins, or DNA. These effects of flavonoids appear to be concentration- and structure-dependent^66^. For instance, glycosylation tends to reduce the in vitro antioxidant activity compared to the corresponding aglycone for several flavonoids, including baicalein and baicalin^66,72^. Notably, baicalin has been reported to have a cytotoxic effect in Jurkat cells, where it stimulated ROS production, leading to caspase-3 activation and apoptosis via the mitochondrial pathway^65^. Furthermore, in human lymphocytes, Yen et al. described an elevation in the amount of superoxide anions and lipid peroxidation products in proportion to an increased concentration of several flavonoids. These substances were also able to induce DNA strand breaks through the formation of hydroxyl radicals in a concentration-dependent manner^73^. Similar concentration-dependent prooxidant and DNA-damaging effects were also observed in rat liver microsomes and human leukocytes^74,75^.

The concentration of the inflammatory cytokines, IL-6 and IL-8 did not change significantly compared to the control cells after 24 h. However, in the combination treatments, the lowest baicalin treatment increased both the IL-6 and IL-8 levels, and the middle baicalin concentration increased the amount of IL-8 when administered together with the lower DON concentration. Furthermore, when combined with the higher DON treatment, both the lowest and middle baicalin elevated the IL-6 levels. The dose-dependent dual effects of DON on the immune system have already been described previously: at low concentrations, it exerts immunostimulatory, while at higher doses, it displays immunosuppressive effects^7,31,33^. Thus, these results suggest that DON, at certain concentrations and combined with baicalin, may have an immunostimulatory effect in the liver cells after 24 h. However, after prolonged exposure, both DON concentrations decreased the IL-8 levels, which is consistent with the theory of an immunosuppressive effect following extended DON exposure. This immunosuppression could be the result of inhibition of the toll-like receptor 4 (TLR4)/NF-kappaB/nucleotide-binding and oligomerization domain-like receptor (NLRP3) pathway by the effect of DON^76^. TLR4 is a key immunomodulator of macrophages, while NLRP3 is an inflammasome. Suppression of the NLRP3 inflammasome by certain toxins, mainly through the regulation of cellular redox status and TLR4 expression, has been linked to immunosuppression^76,77^.

The anti-inflammatory effects of baicalin became more apparent after 48 h. Specifically, IL-6 levels were lowered by the middle baicalin treatment, while IL-8 levels were reduced by the highest applied baicalin concentration. These effects of baicalin are well-documented and are likely mediated through the suppression of the transcription factor NF-κB, leading to the reduced production of pro-inflammatory cytokines^36,37^.

The third inflammatory parameter, IFN-γ, decreased after 24 h at the highest baicalin concentration, consistent with the above-mentioned anti-inflammatory properties of baicalin^36,37^. In addition, when combined with the lower DON treatment, both the middle and the highest baicalin concentrations had a reducing effect. In contrast, the amount of IFN-γ was significantly elevated by the combination of the higher DON and the middle baicalin concentration. This increase is most likely due to the dose-dependent immunostimulant effect of DON and the prooxidant effect of baicalin at certain concentrations. By acting as a prooxidant, baicalin stimulates ROS production in cells, leading to inflammation via the activation of the redox-sensitive NF-κB^29,65,73^. Thus, the combination of these effects could explain the increased levels of IFN-γ. Subsequently, the cells adapted to both DON and baicalin treatments, as shown by the absence of significant changes in the case of the 48 h treatment.

## Conclusion

In summary, these results show that DON reduced cellular metabolic activity, primarily through the induction of oxidative stress, but this effect was not cytotoxic. Baicalin, on the other hand, had a beneficial influence on the viability of the cells and was able to mitigate the adverse effects of DON, particularly by alleviating oxidative stress. This study also highlighted the dual impact of DON on cellular immunity, where low concentrations of the toxin appeared to stimulate, while higher concentrations suppressed the inflammatory cytokine production. However, a potential prooxidant activity of baicalin – particularly after prolonged exposure—was also observed. Furthermore, after 48 h, the cells adapted to the treatments, and defence mechanisms were triggered in order to restore cellular homeostasis. While the applied DON and baicalin concentrations in this study are higher than the supposed tissue levels associated with average feed contamination, they are consistent with concentrations used in previous in vitro studies and are suitable for monitoring the molecular mechanisms linked to DON and baicalin. Although this is a limitation of the study, particularly regarding future in vivo experiments, using a broader range of concentrations enables a more comprehensive understanding of cellular processes. The above-mentioned findings emphasise the complex effect of DON on the cellular oxidative homeostasis and inflammatory processes, with baicalin showing promise as a potential protective agent against these effects. Nonetheless, further research is needed to fully elucidate the mechanisms behind the prooxidant activity of baicalin and its overall role in cellular defence.

## Supplementary Information

Supplementary Information 1.

Supplementary Information 2.

Supplementary Information 3.

## Data Availability

All data generated or analyzed during this study are included in this published article (and its Supplementary Information files).
