# Supplementary figures and images for: Protective effects of baicalin against deoxynivalenol-induced oxidative and inflammatory damage in chicken-derived hepatic 3D cell cultures
Source: Sci Rep. 2025 Apr 1;15:11180. doi: 10.1038/s41598-025-95868-0 (PMC11962109; doi:10.1038/s41598-025-95868-0)

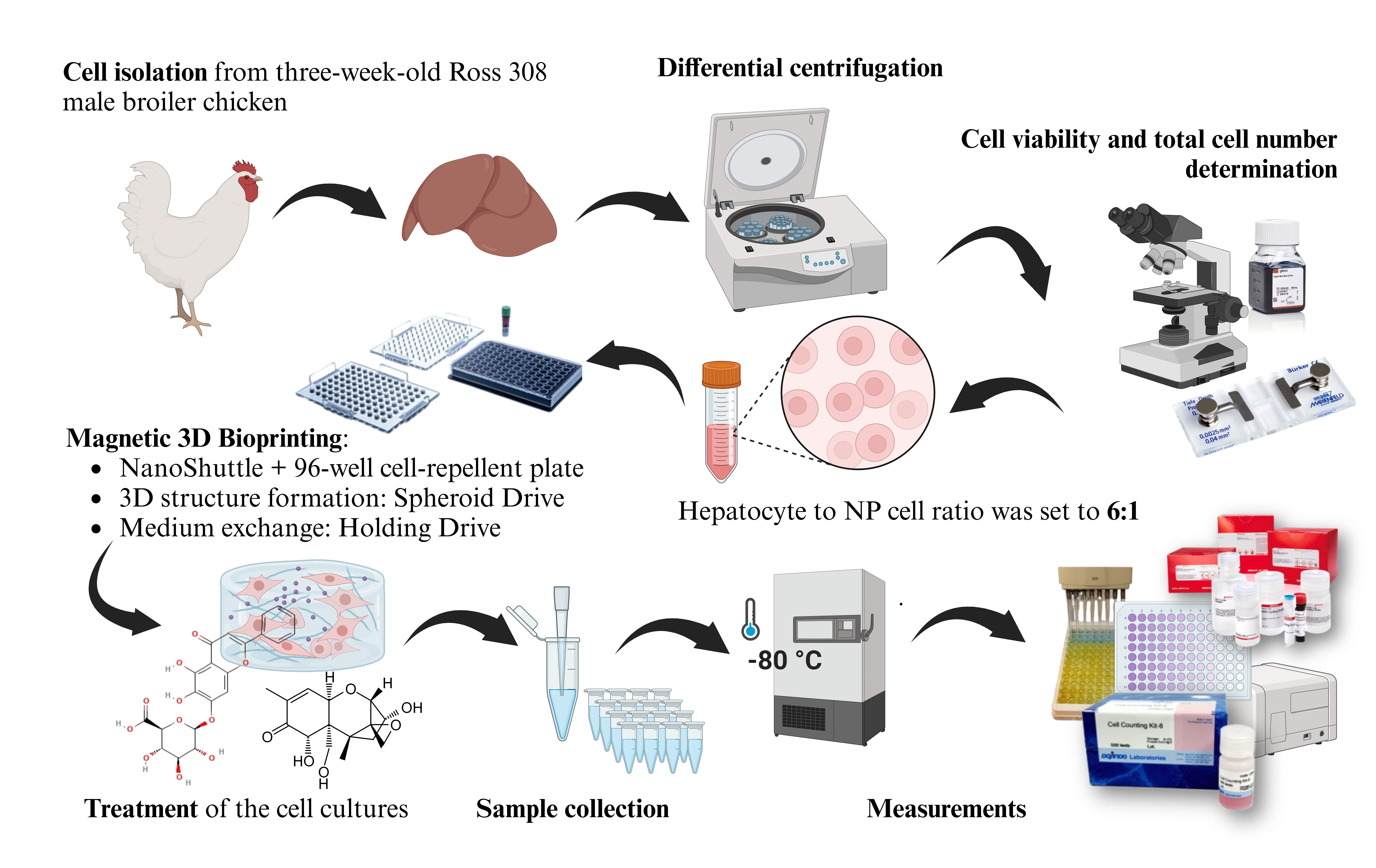

Supplement: Supplementary file 1 — Supplementary Information 1. [file 41598_2025_95868_MOESM1_ESM.png]
