# Supplementary material for: Protective effects of baicalin against deoxynivalenol-induced oxidative and inflammatory damage in chicken-derived hepatic 3D cell cultures
Source: Sci Rep. 2025 Apr 1;15:11180. doi: 10.1038/s41598-025-95868-0 (PMC11962109; doi:10.1038/s41598-025-95868-0)
Supplement: Supplementary file 2 — Supplementary Information 2. [file 41598_2025_95868_MOESM2_ESM.docx]

**Protective effects of baicalin against deoxynivalenol-induced oxidative and inflammatory damage in chicken-derived hepatic 3D cell cultures**

Vörösházi, Júlia^1^*, Mackei, Máté^1,2^, Sebők, Csilla^1^, Tráj, Patrik^1^, Márton, Rege Anna^1,2^, Neogrády, Zsuzsanna^1^, Mátis, Gábor^1,2^

^1^Division of Biochemistry, Department of Physiology and Biochemistry, University of Veterinary Medicine, 1078 Budapest, Hungary

^2^National Laboratory of Infectious Animal Diseases, Antimicrobial Resistance, Veterinary Public Health and Food Chain Safety, University of Veterinary Medicine, 1078 Budapest, Hungary

[*voroshazi.julia@univet.hu](mailto:*voroshazi.julia@univet.hu)

**Supplementary Table 1.** Means of the different measurements with the corresponding SEM and the significance of differences. Cells treated with baicalin or DON alone were compared to the control group, while the combination treatment groups were compared to the corresponding DON treatment group. Differences in the means of the combination treatments and the control group are also included in the table. Control: cells without DON or BAI exposure; BAI: cells treated with BAI; DON: cells treated with DON; DON+BAI: cells treated with the combination of DON and BAI. See Table 1 for detailed concentrations. * p < 0.05; ** p < 0.01; *** p < 0.001.

**Supplementary Figure 1**. Schematic representation of cell isolation, treatments, and collection of samples. The figure was prepared using the online software <https://www.biorender.com/>.
